# Metabolomic Analysis Reveals That the Mechanism of Astaxanthin Improves the Osteogenic Differentiation Potential in Bone Marrow Mesenchymal Stem Cells

**DOI:** 10.1155/2020/3427430

**Published:** 2020-03-25

**Authors:** Guangfeng Zhao, Huiming Zhong, Taiwen Rao, Zhijun Pan

**Affiliations:** ^1^Department of Emergency, The Second Affiliated Hospital, Zhejiang University School of Medicine, Zhejiang University, Hangzhou 310009, China; ^2^Department of Orthopaedics, The Second Affiliated Hospital, Zhejiang University School of Medicine, Zhejiang University, 88 Jiefang Road, Hangzhou 310009, China

## Abstract

At present, little research has been done on the metabolic phenotype of the differentiation of mesenchymal stem cells (MSCs) into osteoblasts. In this study, the effect of astaxanthin on improving osteogenic differentiation potential of mesenchymal stem cells was studied by metabolomics. Results showed that L-methionine, L-tyrosine, and 2-hydroxycinnamic acid were upregulated in MSCs treated with astaxanthin, while L-lysine, L-pipecolic acid, L-histidine, L-arginine, D-fructose, and L-aspartic acid were downregulated in samples treated with astaxanthin. In addition, astaxanthin exhibited a significant dose-dependent relationship with these markers. Metabolic pathway enrichment analysis revealed that AST mainly regulated phenylalanine metabolism; phenylalanine, tyrosine, and tryptophan biosynthesis; and pantothenate and CoA biosynthesis during the process of osteogenic differentiation of MSCs. Furthermore, the staining results showed that astaxanthin could actively promote the osteogenic differentiation of mesenchymal stem cells. These findings clearly indicate that astaxanthin plays an important role in inducing osteogenic differentiation of mesenchymal stem cells. In addition, the changed metabolites can be used to monitor the differentiation process.

## 1. Introduction

Mesenchymal stem cells (MSCs) are multifunctional cells widely used in muscle and skeletal tissue regeneration and currently account for the largest share of the global tissue engineering market [1, 2]. MSCs derived from fetal tissues have become an attractive alternative to adult mesenchymal stem cells. Because of their high proliferation potential, they have become a promising source of cells for personalized bone transplantation [3, 4]. Mesenchymal stem cells undergo unique metabolic changes during their differentiation into osteoblasts. At present, there is little information about the metabolic phenotype of the process of osteogenesis. Astaxanthin (AST), a lipid-soluble red-orange carotenoid pigment discovered in 1938, was originally used as the sole pigmentation in aquaculture. Then, AST was used as a food supplement due to its antioxidant physiological properties as the precursor of vitamin A [5]. At present, AST research is increasing due to its promotion of human health [6]. AST exhibits several of the common physiological and metabolic activities assigned to carotenoids [7, 8]. AST can be employed in the treatment of OA [9]. AST reduces the expression of MMP-1, MMP-3, and MMP-13 in osteoarthritis, rheumatoid arthritis, and osteoporosis and the phosphorylation of chondrocytes induced by MAPK p38, ERK1/2, and IL-1beta [10]. AST can also enhance the proliferation and differentiation of osteoblasts in neural stem cells [11].

Metabolomics provides a global analysis of cell metabolism and has been proven to be a useful tool for monitoring cell culture [12]. Metabolomics can be used for the study of the physiology of stem cell metabolism to clarify the metabolic network state [13], by use of multivariate analysis to explain the correlation between pathways, which is neglected by traditional enzyme activity tests that focus on a limited number of enzymes and the analysis of specific metabolites, and it also has been used to identify small molecules secreted by ESCs when exposed to sodium valproate [14] and steroid hormones [15] and to monitor cartilage formation in MSCs [16]. Recent studies on the metabolism of MSCs, mainly based on extracellular metabolites, have shown that differentiated MSCs exhibit a specific metabolic phenotype. However, a specific marker of osteogenic differentiation of mesenchymal stem cells was not identified.

In our study, LCMS was used to detect the metabolite changes of mesenchymal stem cells (MSCs) during the differentiation of osteoblasts induced by AST. The results showed that mesenchymal stem cells exhibited a specific metabolite change related to the differentiation. This study shows that astaxanthin has the ability to induce osteogenic differentiation of mesenchymal stem cells. It is feasible to monitor the differentiation of mesenchymal stem cells by using cell-specific metabolic markers identified by LCMS.

## 2. Materials and Methods

### 2.1. Experimental Procedures

DMEM medium, fetal bovine serum, penicillin, and streptomycin were purchased from Gibco (Gibco, Life Technologies, UK). Trypsin, beta-glycerophosphate, 3-isobutyl-1-methylxanthine (IBMX), dexamethasone sodium phosphate, L-ascorbic acid, and insulin were purchased from Sigma-Aldrich Chemicals (St. Louis, MO, USA). CD34, CD44, CD45, and CD90 antibodies were obtained from BD Biosciences. Astaxanthin was purchased from Sigma-Aldrich Chemicals (St. Louis, MO, USA) and was made a stock solution with dimethyl sulfoxide (DMSO) and then was diluted to 1 *μ*g/ml and 10 *μ*g/ml.

### 2.2. Isolation and Culture of BMSCs

Rats were anesthetized with 2% pentobarbital, and 75% ethanol solution was used for a 5 min whole body disinfection. The femur and tibia were removed under aseptic conditions and cleaned 3 times with PBS. The epiphyses of the femur and tibia were removed to expose the bone marrow cavity. The bone marrow was washed out with DMEM medium supplemented with penicillin and streptomycin, resuspended repeatedly into a single cell suspension, and then centrifuged at 1000 rpm for 5 min. The supernatant was discarded, and the cells were inoculated into a 25 cm [3] culture flask at a concentration of 1 × 10^9^/l.

### 2.3. Cultivation, Purification, and Passage of BMSCs

After 48 h, the medium was completely replaced and thereafter replaced with fresh medium every 3 days. The cells were digested with 0.25% trypsin and subcultured in a ratio of 1 : 2 when the cells covered the bottom of the flask and fused into a single layer with a density of 70%~80%. Cells were further cultured with the same culture medium at 37°C in an environment of 21% O_2_ and 5% CO_2_. The culture medium was changed twice a week, and the cells were passaged before reaching 80% confluence. Cells between passages 4 and 6 were used for subsequent experiments. For osteogenic differentiation, the culture medium was supplemented with 1 *μ*g/ml and 10 *μ*g/ml AST (Sigma-Aldrich, UK).

Bone marrow mesenchymal cells (BMSCs), from SD rats, that were growing well were cultured for 2 generations and inoculated into a 96-well culture plate. When the density of the BMSCs was 5∗10^3^/well, the culture plate was placed in a CO_2_ incubator. Aliquots were taken daily for the CCK-8 assay. The wavelength of 450 nm was selected, and the optical absorption value of each well was measured by ELISA. The cell growth curve was drawn on the longitudinal axis with the time as the transverse axis and the light absorption value as the longitudinal axis. The cell concentration was adjusted to 1∗10^9^/l. The cell cycle was detected by flow cytometry after a 30 min incubation with 100 *μ*l RNAase and 400 *μ*l PI stain in a water bath at 37°C.

### 2.4. Identification of BMSCs

The cells were digested by 0.25% trypsin and centrifuged at 4°C at 1000 rpm for 5 min. The cells were washed 3 times with PBS (containing 1% BSA) and then counted. Monoclonal antibodies CD34, CD44, CD45, and CD90 were then added to the tubes. The cells were incubated on dry ice for 45 min and washed with PBS (containing 1% BSA) three times to remove the unbounded antibody. The cells were suspended with 500 *μ*l of PBS (containing 1% BSA) and analyzed by flow cytometry.

### 2.5. Differentiation of BMSCs In Vitro

When the cell density reached 80%, the following inducers were added to the complete culture medium of each induction well: (1) adipocyte inducers (1 mmol/l dexamethasone, 10 mg/l insulin, 50 mmol/l IBMX, and 0.2 mmol/l indomethacin) and (2) osteoblast inducers (1 mmol/l dexamethasone, 1 mmol/l beta-glycerophosphate, and 50 mmol/l ascorbic acid). The inducting medium in each induction well was changed 2 times a week, and the cell culture wells without inducing medium were used as controls. After 21 days of induction, the effect of AST on BMSC culture mineralization was evaluated using Alizarin Red Staining (ARS). And cells were stained for 15 min with 0.01% ARS (3,4-dihydroxy-9,10-dioxo-2-nthracenesulfonic acid sodium salt, from Sigma-Aldrich, St. Louis, MO) dissolved in 70% ethanol. Acetic acid (10%) (Sigma-Aldrich, UK) was added to the ARS-stained culture, and the suspension was vortexed and heated to 85°C for 10 min. Subsequently, the pH was adjusted between 4.1 and 4.5 with 10% ammonium hydroxide (Sigma-Aldrich, UK), and the absorbance values were read at 405 nm with an ELISA reader (ELx808, BioTek, UK). Cell nuclei were evident due to hematoxylin counterstaining. Images were captured using a digital color camera (Nikon D1) mounted on the microscope. The end results were quantified as described in this paper [17].

For the sample selected for LCMS analysis, the growth medium was removed, cells were washed with sterile 1x PBS and quenched with 1 ml ice-cold methanol, and the mixture was transferred to 5 ml tubes. And the mixture was quickly vortexed and then centrifuged at 12,000 rpm for 10 min at 4°C. The supernatants were transferred to the injection vial for LCMS analysis.

### 2.6. Metabolomic Analysis

LCMS was performed using a binary high-performance liquid chromatography system (Agilent 1290 series) connected to an electrospray ionization time-of-flight mass spectrometer (Agilent 6545). Chromatographic separation was carried out on a Waters ACQUITY UPLC BEH C18 analytical column (2.1 × 100 mm, 1.7 *μ*m, pore size 130 Å; Waters Co.). Solvent A consisted of water with 0.1% (*v*/*v*) formic acid, and solvent B consisted of ACN with 0.1% (*v*/*v*) formic acid. The gradient was as follows: 0-2 min, 2% B; 2-15 min, 2-95% B; and 15-20 min, 95% B. The flow rate was 350 *μ*l/min.

### 2.7. Data Processing and Statistical Analyses

Raw data were converted to a common (mz. data) format by Agilent MassHunter Qualitative Analysis B.08.00 software (Agilent Technologies, USA). The XCMS package in R software was used for peak extraction and peak matching. Then, the data were normalized with an internal standard. A table containing the sample name, *m*/*z*-RT pair, and peak area was obtained, and then, the data was analyzed with principal component analysis (PCA) and partial least squares discriminant analysis (PLS-DA) by SIMCA-P 13.0 software (Umetrics, Umea, Sweden). The metabolites were identified based on retention time and accurate mass matching to a standard library or accurate mass matching to the human metabolome database (HMDB). The mass accuracy tolerance window was set at 30 ppm for the database search. Metabolite set enrichment analysis and pathway analysis were based on MetaboAnalyst (http://www.metaboanalyst.ca) using the Homo sapiens pathway library.

## 3. Results

### 3.1. Morphological Observation of BMSC Primary Cultures

**Figure 1:**
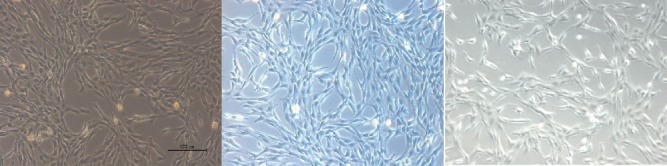
Morphological observation of BMSCs.

**Figure 2:**
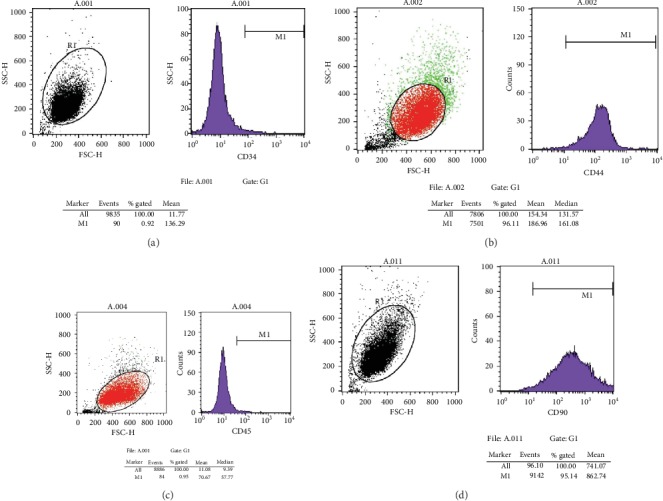
Expression of markers in BMSCs of the third generation. (a) For the expression of CD34, the positive rate was 0.92%; (b) for the expression of CD44, the positive rate was 96.11%; (c) for the expression of CD45, the positive rate was 0.95%; and (d) for the expression of CD90, the positive rate was 95.14%.

**Figure 3:**
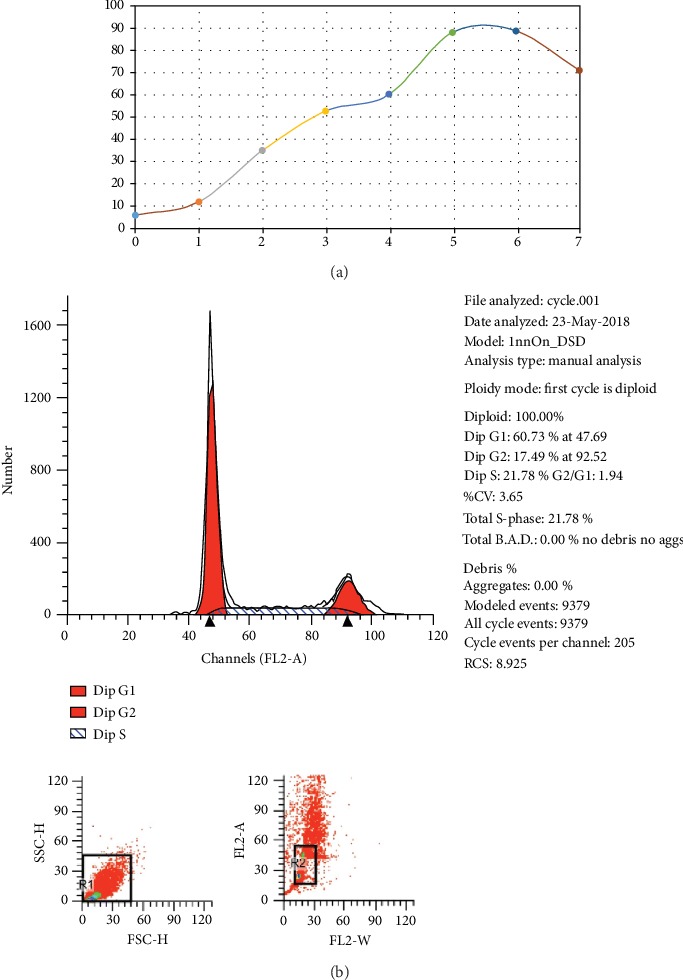
Cell cycle and growth curve of BMSCs. (a) Growth curve of BMSCs: the growth curve of cells on the 3rd to 6th day is basically linear, which indicates that this period is the logarithmic growth period of cells. After 7 days, the curve became smooth and cell proliferation slowed down. (b) Cell cycle: the results of cell cycle analysis by flow cytometry showed that 60.73% of BMSCs were in the G0/G1 phase, 17.49% in the G2/M phase, and 21.78% in the S-phase, indicating that BMSCs had strong ability to divide and proliferate.

After inoculating bone marrow cells into culture flasks, the cells suspended in the culture medium were round and varied in size. After 24 h, some cells began to adhere to the wall, exhibiting fusiform or polygonal shapes, as shown in Figure 1. The results of flow cytometry showed that CD44 was uniformly expressed in 3rd-generation BMSCs (96.11%), and the rate of CD90 positivity was 95.14%; however, CD34 and CD45 were negative, with positivity rates of 0.92% and 0.95%, respectively, as shown in Figure 2. Differences in the osteogenic efficacy of AST are associated with distinct metabolic transitions. Flow cytometry analysis showed that 60.73% of BMSCs were in the G0/G1 phase, 17.49% were in the G2/M phase, and 21.78% were in the S-phase, indicating that BMSCs had a strong ability to divide and proliferate, as shown in Figure 3.

### 3.2. AST-Induced Osteogenesis of MSCs

**Figure 4:**
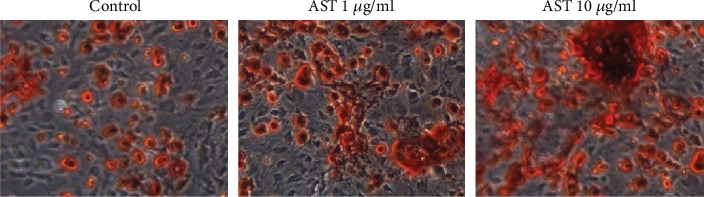
Alizarin Red S over 21 days of differentiation. Con: control group, medium+vehicle; AST: astaxanthin group. The mineralization of the AST groups was higher than that of the control group. The area of red staining in the 10 *μ*g/ml dose group and the 1 mg/ml dose group was larger than that of the control group, and the area of the 10 *μ*g/ml dose group was larger than that of the 1 mg/ml dose group.

The ability of AST to induce osteogenic differentiation was evaluated by Alizarin Red Stain. The result showed bright red staining in the AST groups, and the mineralization of the AST groups was higher than that of the control group, as shown in Figure 4.

### 3.3. Significant Metabolite Changes and Metabolic Pathway Analysis

**Figure 5:**
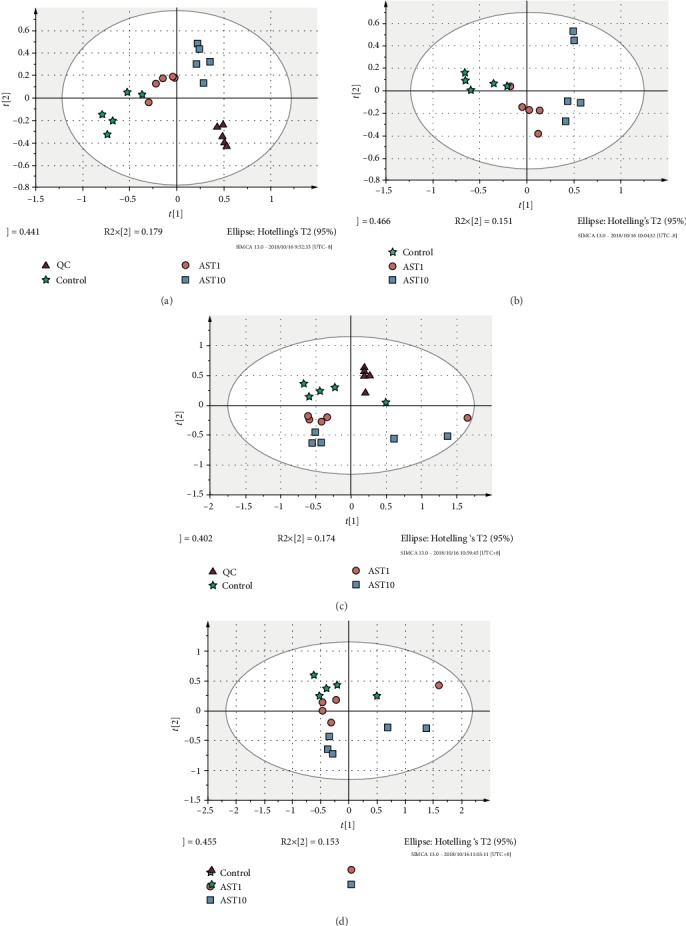
Principal component analyses of AST1, AST10, and control group cells. ▲ represents QC samples, ★ represents control samples, ● represents AST-1 *μ*g/ml samples, and ■ represents AST-10 *μ*g/ml samples.

**Figure 6:**
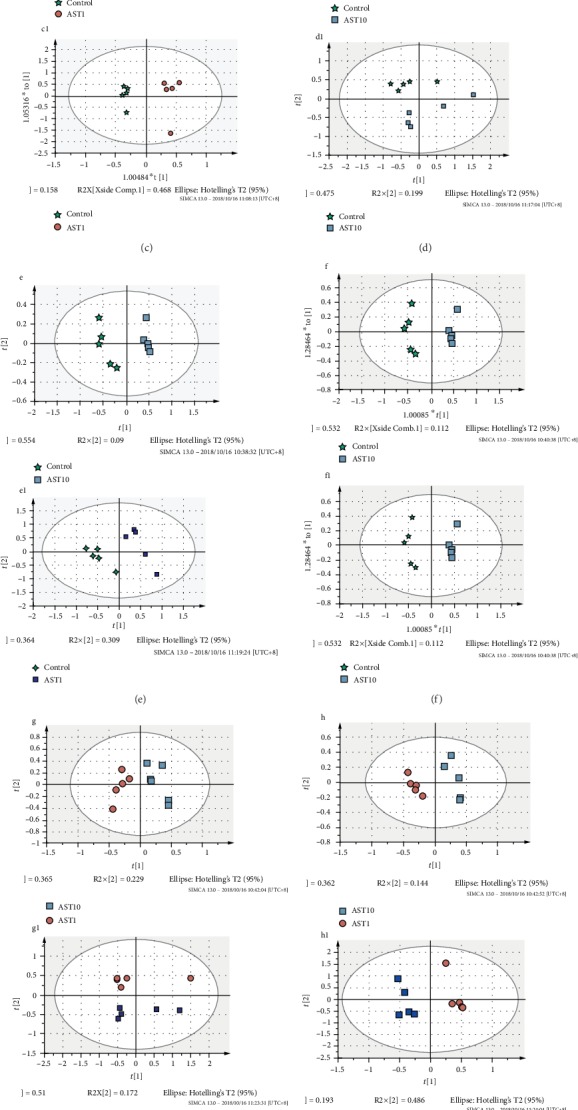
PCA, PLS-DA, and OPLSDA of AST1, AST10, and control group cells. (a–c) Positive ion mode: AST1 vs. control; (a1–c1) negative ion mode: AST1 vs. control; (d–f) positive ion mode: AST10 vs. control; (d1–f1) negative ion mode: AST10 vs. control; (g–i) positive ion mode: AST10 vs. AST1; (g1–i1) negative ion mode: AST10 vs. AST1. ★ represents control samples, ● represents AST-1 *μ*g/ml samples, and ■ represents AST-10 *μ*g/ml samples.

The effects of AST on the metabolism of mesenchymal stem cells during osteogenesis were evaluated by metabolic analysis, and the metabolic differences among different doses of AST were compared. The results showed that the metabolism of undifferentiated cells and differentiated cells was significantly different, and different doses of AST-induced differentiation also showed different metabolic profiles. As shown in the score plots of the PCA analysis (Figure 5), the AST1 group, AST10 group, and control group are separated from each other and clustered in different areas. The PLS-DA models (Figure 6) also showed separation between the two experimental groups (AST1/C, AST10/C, and AST10/AST1). Volcano plot analyses, which combined the fold change (FC) and *p* values from the *t*-tests, were also used to identify the unique metabolites that separated the two groups (control and AST groups). Metabolites with a *p* < 0.05 and an FC of either >1.5 or <0.67 (i.e., >±50% change) were considered significant (red dots). Using these thresholds, only 25 correlated markers (Table 1) were significantly changed in response to AST treatment. L-Methionine, L-tyrosine, and 2-hydroxycinnamic acid were upregulated in samples treated with astaxanthin, while L-lysine, L-pipecolic acid, L-histidine, L-arginine, D-fructose, and L-aspartic acid were downregulated in samples treated with astaxanthin. Metabolic pathway enrichment analysis revealed that AST mainly regulated phenylalanine metabolism; phenylalanine, tyrosine, and tryptophan biosynthesis; and pantothenate and CoA biosynthesis during the process of osteogenic differentiation of MSCs (Table 2).

## 4. Discussion

It is recommended that metabolic profiles be used to monitor the differentiation of stem cells and to establish quality control standards for biotechnology processes and products. The study of metabolomics has shown the success of osteogenic differentiation of bone marrow mesenchymal stem cells stimulated by dexamethasone [18]. Surrati et al. monitored the osteogenesis of mouse mesenchymal stem cells treated with dexamethasone. It was found that the TCA cycle and glycerol derivatives were increased in the medium [19]. However, these studies were limited by the detection of extracellular metabolites that provided only an approximation of intracellular metabolism [20].

In the present study, MSC differentiation induced by AST was characterized by cellular metabolomics. L-Methionine, L-tyrosine, PC(P-17:0/0:0), and oleic acid were upregulated in samples treated with astaxanthin, while L-lysine, L-pipecolic acid, L-histidine, L-arginine, D-fructose, and palmitic acid were downregulated in samples treated with astaxanthin. In addition, there was a significant dose-dependent relationship with these markers. The activation of pathways is related to amino acid metabolism, fatty acid biosynthesis, and lipid metabolism during the osteogenic differentiation. These observations emphasize the fact that metabolism changes significantly during (osteogenic) differentiation. These amino acids are fundamental factors in nutrition as building blocks for biomass components, such as DNA, RNA, and proteins [21]. Our results indicated that BMSCs consumed L-lysine, L-histidine, L-arginine, L-valine, L-leucine, and L-phenylalanine. The observed metabolic pattern is proposed to be representative of the basic nutritional requirements for BMSCs in culture.

In addition, fatty acids have previously been reported to affect cell survival. Saturated fatty acids have specifically been reported to induce death in many cell types, including BMSCs [22, 23]. In the present study, palmitic acid was decreased in the AST-treated group, while the level of oleic acid was higher in the AST-treated group. Fatty acids can regulate flux through energy metabolic pathways and may thereby regulate cell survival. A previous study showed that oleic acid could prevent palmitic acid-induced BMSC death [24]. These results indicate that the AST treatment may increase the level of unsaturated fatty acids for the proliferation and differentiation of BMSCs. Interestingly, in our study, phosphocholine was increased in the AST-induced group, and the increased phosphocholine level during chondrogenesis was also found in the process of differentiation of hMSCs into chondrocytes [25, 26]. The above results show that the phospholipid level in osteoblasts is higher than that in nonproliferation bone marrow mesenchymal stem cells, so it may be a marker of cell differentiation and proliferation.

## Figures and Tables

**Table 1:** Significant changes in metabolites of AST1 vs. control, AST10 vs. control, and AST10 vs. AST1.

*m*/*z*	RT (min)	FC (AST10/C)	FC (AST1/C)	FC (AST10/AST1)	Name	Formula
147.1163	0.69	0.27	0.53	0.51	L-Lysine	C_6_H_14_N_2_O_2_
130.0839	0.72	0.22	0.61	0.36	L-Pipecolic acid	C_6_H_11_NO_2_
156.0744	0.73	0.14	0.26	0.54	L-Histidine	C_6_H_9_N_3_O_2_
175.1169	0.76	0.24	0.23	1.07	L-Arginine	C_6_H_14_N_4_O_2_
179.0581	0.78	0.12	0.27	0.44	D-Fructose	C_6_H_12_O_6_
132.0328	0.79	0.11	0.15	0.74	L-Aspartic acid	C_4_H_7_NO_4_
282.2554	11.01	2.15	1.21	1.77	Oleic acid	C_5_H_7_NO_3_
118.0892	0.95	0.36	0.56	0.65	L-Valine	C_5_H_11_NO_2_
148.0455	1.08	75.49	3.39	22.27	L-Methionine	C_5_H_11_NO_2_S
182.0824	1.17	82.51	1.87	44.18	L-Tyrosine	C_9_H_11_NO_3_
165.0541	1.18	15.82	1.47	10.76	2-Hydroxycinnamic acid	C_9_H_8_O_3_
130.0882	1.26	0.15	0.63	0.24	L-Leucine	C_6_H_13_NO_2_
256.2399	10.82	0.11	0.59	0.18	Palmitic acid	C_9_H_7_NO
557.3051	7.57	2.10	1.55	1.35	PC(P-17:0/0:0)	C_25_H_52_NO_6_P
136.0766	1.85	0.12	0.46	0.26	2-Phenylacetamide	C_8_H_9_NO
209.0926	2.17	0.28	0.49	0.59	Kynurenine	C_10_H_12_N_2_O_3_
149.0601	2.19	0.19	0.52	0.36	*trans*-Cinnamic acid	C_9_H_8_O_2_
166.0915	2.19	0.23	0.45	0.52	L-Phenylalanine	C_9_H_11_NO_2_
218.1052	2.78	0.15	0.39	0.40	Pantothenic acid	C_9_H_17_NO_5_
279.1355	3.51	0.10	0.35	0.28	Prolyl-tyrosine	C_14_H_18_N_2_O_4_
146.0628	4.09	0.17	0.34	0.50	4-Formyl indole	C_9_H_7_NO
205.1034	4.09	0.20	0.29	0.71	L-Tryptophan	C_11_H_12_N_2_O_2_
118.0676	4.09	0.23	0.28	0.83	Indole	C_8_H_7_N
493.3525	8.78	2.33	1.50	1.56	PC(21:4(6Z,9Z,12Z,15Z)/0:0)	C_29_H_52_NO_7_P

**Table 2:** Pathway analysis of different significant changes in metabolites of AST1 vs. control, AST10 vs. control, and AST10 vs. AST1.

Pathway name	Match status	*p*	-Log(*p*)	Holm *p*	FDR	Impact
Aminoacyl-tRNA biosynthesis	10/75	1.2581*E*‐10	22.796	1.0065*E*‐8	1.0065*E*‐8	0.11268
Nitrogen metabolism	5/39	1.4452*E*‐5	11.145	0.0011417	5.7808*E*‐4	6.7*E*‐4
Phenylalanine metabolism	5/45	2.9653*E*‐5	10.426	0.0023129	7.9075*E*‐4	0.32518
Phenylalanine, tyrosine, and tryptophan biosynthesis	4/27	6.6065*E*‐5	9.6249	0.005087	0.0013213	0.008
Pantothenate and CoA biosynthesis	3/27	0.0014641	6.5265	0.11127	0.021742	0.18014
Beta-alanine metabolism	3/28	0.0016306	6.4188	0.1223	0.021742	0.0
Biotin metabolism	2/11	0.0038039	5.5717	0.28149	0.043473	0.20325
Valine, leucine, and isoleucine biosynthesis	2/27	0.02232	3.8023	1.0	0.2232	0.0265
Tryptophan metabolism	3/79	0.029564	3.5212	1.0	0.24582	0.16587
Lysine biosynthesis	2/32	0.030727	3.4826	1.0	0.24582	0.09993
Valine, leucine, and isoleucine degradation	2/40	0.046347	3.0716	1.0	0.33707	0.02232

## Data Availability

The datasets used and analyzed during the current study are available from the corresponding author upon reasonable request.
